# An open label, randomized phase 2 trial assessing the impact of food on the tolerability of abemaciclib in patients with advanced breast cancer

**DOI:** 10.1007/s10549-022-06690-5

**Published:** 2022-08-01

**Authors:** Elgene Lim, Frances Boyle, Meena Okera, Sherene Loi, Sema Sezgin Goksu, Gertjan van Hal, Sonya C. Chapman, Jonathon Colby Gable, Yanyun Chen, Gregory L. Price, Anwar M. Hossain, M. Corona Gainford, Meritxell Bellet Ezquerra

**Affiliations:** 1 Garvan Institute of Medical Research, St. Vincent’s Clinical School, University of New South Wales, 370 Victoria Street, Darlinghurst, NSW Australia; 2 Mater Hospital, North Sydney, NSW Australia; 3 Adelaide Cancer Centre, Kurralta Park, SA Australia; 4 Peter MacCallum Cancer Centre, Melbourne, VI Australia; 5 Akdeniz University Medical Faculty, Antalya, Turkey; 6 Eli Lilly and Company, Utrecht, Netherlands; 7 Eli Lilly and Company, Windlesham, Surrey UK; 8 Eli Lilly and Company, Indianapolis, IN USA; 9 Hospital Universitario Vall d’Hebron and Vall d’Hebron Institute of Oncology (VHIO), Barcelona, Spain

**Keywords:** CDK4/6 inhibitor, Tolerability, Breast cancer

## Abstract

**Purpose:**

Abemaciclib, a CDK4 & 6 inhibitor, is indicated for advanced breast cancer treatment. Diarrhea is a frequently associated adverse event of abemaciclib. The study objective was to investigate if food intake impacts local gastrointestinal toxicity.

**Methods:**

This Phase 2 study (I3Y-MC-JPCP, NCT03703466) randomized 72 patients 1:1:1 to receive abemaciclib 200 mg monotherapy twice daily (1) with a meal, (2) in a modified fasting state or (3) without regard to food. Primary endpoints included: incidence of investigator assessed severe (≥ Grade 3), prolonged (> 7 days) Grade 2 diarrhea, treatment discontinuation, dose modifications, and loperamide utilization during the first 3 cycles of treatment. Patient outcomes were captured via a daily electronic diary. Pharmacokinetics (PK) are reported.

**Results:**

Incidence of investigator assessed severe diarrhea (Grade ≥ 3) was 1.4% (1 patient in Arm 1). Median duration of Grade 3 diarrhea was 1 day by both investigator assessment (1 patient in Arm 1) and patient-reported assessment (1 patient each in Arms 1 and 3). Median duration of investigator-assessed Grade 2 diarrhea was 2 days overall. No patient discontinued treatment due to diarrhea. Nine patients (12.7%) had a dose reduction, and 7 patients (9.9%) had a dose omission due to diarrhea. Ninety-four percent of patients used loperamide at least once. Abemaciclib PK was comparable across the 3 arms.

**Conclusion:**

The results suggest that diarrhea incidence associated with abemaciclib was unrelated to timing of food intake, was predominantly low grade, of short duration and well managed with loperamide and dose modifications.

**Supplementary Information:**

The online version contains supplementary material available at 10.1007/s10549-022-06690-5.

## Introduction

Abemaciclib is an oral, selective, and potent inhibitor of cyclin-dependent kinases 4 and 6 (CDK4 & 6) dosed twice daily (BID) on a continuous schedule [1]. Resulting from the MONARCH series of clinical trials, abemaciclib is approved as monotherapy and in combination with endocrine therapy (ET) for the treatment of patients with hormone receptor-positive (HR +), human epidermal growth factor receptor 2-negative (HER2-) advanced breast cancer (ABC) [2].

MONARCH 1, a single-arm Phase 2 study of abemaciclib 200 mg BID monotherapy in patients with refractory HR + , HER2- ABC, demonstrated promising clinical activity (objective response rate (ORR) of 19.7% (95% confidence interval [CI]: 13.3–27.5) [3].

MONARCH 2 was a randomized, double-blind, Phase 3 study of abemaciclib 150 mg BID in combination with fulvestrant compared to placebo plus fulvestrant in women with HR + , HER2 − ABC who had progressed following ET therapy [1]. This trial demonstrated significantly improved progression-free survival (PFS) (median 16.4 versus 9.3 months; hazard ratio [HR] [95% CI]: 0.553 [0.449–0.681]; *p* < 0.001) and overall survival (OS) (median 46.7 versus 37.3 months; HR [95% CI]: 0.757 [0.606–0.945]; *p* = 0.01) [1, 4].

MONARCH 3 was a randomized, double-blind Phase 3 study of abemaciclib 150 mg BID in combination with a non-steroidal aromatase inhibitor (NSAI) compared to placebo plus NSAI as initial therapy in women with HR + , HER2 − ABC [5]. Abemaciclib plus NSAI significantly improved PFS (median 28.18 versus 14.76 months; HR [95% CI]: 0.540 [0.418–0.698]; *p* = 0.000002).

**Fig. 1:**
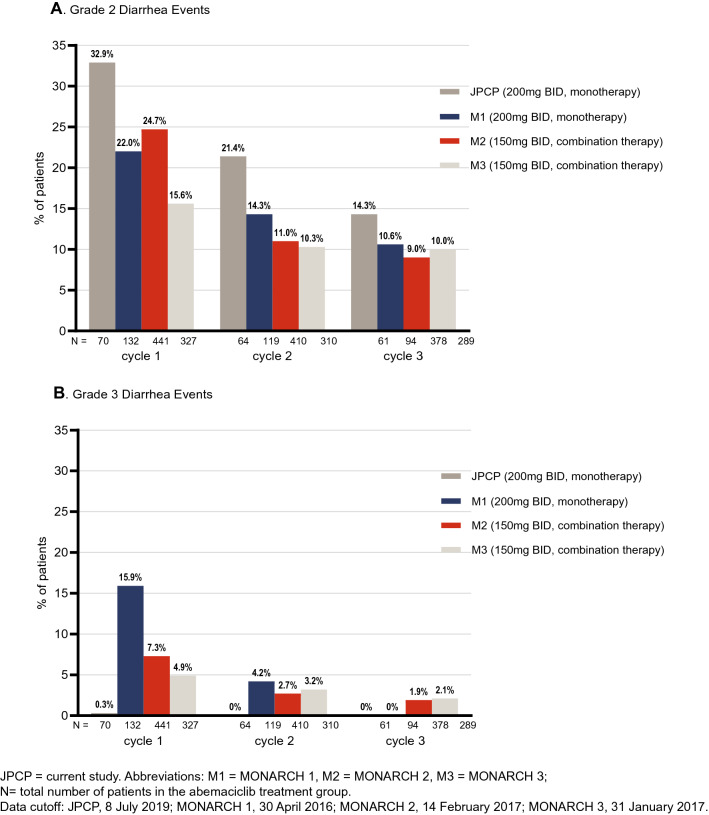
Comparison of investigator-assessed treatment-emergent diarrhea in patients receiving abemaciclib from studies JPCP, MONARCH 1, MONARCH 2, and MONARCH 3

Diarrhea was the most frequently reported treatment-emergent adverse event (TEAE) of any grade in patients prescribed abemaciclib in all three pivotal studies, irrespective of whether abemaciclib was taken as a monotherapy (MONARCH 1 [200 mg BID]: diarrhea = 90.2%) or in combination with ET (MONARCH 2 and MONARCH 3 [150 mg BID]: diarrhea = 87.1% and 82.3%, respectively) [1, 3, 5]. Grade 3 diarrhea was reported in 20% of patients in MONARCH 1, 14% in MONARCH 2 and 10% in MONARCH 3 [3–5]. In all three trials, incidence of Grade 2 and Grade 3 diarrhea was greatest during the first month of treatment and decreased over the remaining cycles of therapy (Fig. 1). The median duration of any grade diarrhea was similar across the 3 trials ranging 6–8 days, with the median duration of Grade 2 diarrhea ranging 8–11 days and Grade 3 diarrhea, 5–8 days [1, 3, 5]. In each study, diarrhea was retrospectively assessed by the investigator at the beginning of each 28-day cycle and graded as per Common Terminology Criteria for Adverse events (CTCAE) criteria [1, 3, 5]. To our knowledge, no patient-reported daily data on abemaciclib and diarrhea have been published to date.

Standardized anti-diarrheal management plans are outlined in the product label [2]. Patients are advised to commence over-the-counter (OTC) antidiarrheal medication such as loperamide at the first sign of loose stools, increase their fluid intake, and contact their physician. If diarrhea has not resolved within 24 h, abemaciclib should be suspended until resolution occurs. Resolution is defined as either a reduction to baseline or Grade 1 (< 4 stools per day increase over baseline). The label also includes detailed guidance for dose modifications and reductions according to severity of diarrhea [2].

Consistent with management guidance, 21%, 19%, and 14% of patients in MONARCH 1, 2, and 3 respectively required dose reductions and 1%, 3%, and 2% respectively discontinued the study drug due to diarrhea [1, 3, 5]. Antidiarrheal use across the studies varied between 61% in MONARCH 1, 76% in MONARCH 2, and 61% in MONARCH 3.

In MONARCH 2 and 3, a paper version of the patient-reported outcomes (PRO) assessment was completed once at baseline, and on treatment; with more frequent collection in earlier cycles, and at the follow-up visit [6, 7]. PRO results from abemaciclib in combination with fulvestrant (MONARCH 2) or in combination with NSAI (MONARCH 3) did not show clinically significant differences in patient-reported global health, functioning, or most symptoms compared to ET alone [6, 7]. Baseline scores were similar between treatment arms in each study. In both trials, diarrhea was the only patient-reported symptom with a statistically significant and clinically meaningful difference between treatment arms. These diarrhea findings were reported in early treatment cycles, consistent with investigator assessments, decreased in later cycles and returned to near baseline levels at the post-therapy follow-up visits [6, 7].

For some drugs, coadministration with food can impact bioavailability and may have clinically significant consequences. In clinical studies, a high-fat, high-calorie meal increased the exposure (AUC) of abemaciclib analytes by 9% and increased C_max_ by 25% [8]. These changes in exposure are not clinically meaningful and abemaciclib is therefore given without regard to food. However, it is possible that taking abemaciclib with food may impact local gastrointestinal toxicity independently of systemic pharmacokinetics (PK) and thus alter drug tolerability. As an example, ingestion of food with nonsteroidal anti-inflammatory drugs (NSAIDs) is often preferred because it reduces local gastrointestinal adverse effects [9]. In order to address this issue and at the request of the U.S. Food and Drug Administration (FDA), this randomized study (I3Y-MC-JPCP) evaluated the impact of coadministration of food on the incidence and tolerability of diarrhea in patients with HR + , HER2- ABC receiving abemaciclib monotherapy.

## Methods

### Study design and patients

JPCP (NCT03703466) was a global, randomized, open-label Phase 2 study evaluating the timing of food intake on the incidence of severe diarrhea (Grade ≥ 3) or prolonged Grade 2 diarrhea (> 7 days duration) when receiving abemaciclib monotherapy 200 mg orally (PO) BID in patients with previously treated HR + , HER2- ABC.

The study was conducted at 15 centers in 5 countries (Australia, Belgium, Russian Federation, Spain and Turkey). It was approved by ethical and local institutional review boards for the participating sites and was conducted according to the Declaration of Helsinki. Patients provided written informed consent prior to trial enrollment. The study was overseen by an ethics review board.

Eligible patients included males and females ≥ 18 years of age with a diagnosis of recurrent, locally advanced, unresectable, or metastatic HR + , HER2- ABC. Patients were required to have an Eastern Cooperative Oncology Group (ECOG) performance status of ≤ 1; a willingness to use an e-diary; and having no prior use of a CKD4 & 6 inhibitor. Patients must have received ≥ 1 chemotherapy regimen and progressed after prior anti-estrogen therapy for ABC. Patients were required to have discontinued all previous treatments for cancer and recovered from the acute effects of therapy. Per protocol, length of time between end of previous treatment and first abemaciclib dose was 14 to 28 days, depending on type of prior treatment. Patients were ineligible if they had a serious concomitant systemic disorder (for example, active infection or a gastrointestinal disorder causing clinically significant symptoms such as nausea, vomiting or diarrhea [such as Crohn’s disease, ulcerative colitis], or profound immune suppression) or a serious preexisting medical condition (for example, history of major surgical resection involving the stomach or small bowel) that, in the opinion of the investigator, would compromise/preclude the patient’s ability to adhere to the protocol.

Patients were randomly assigned 1:1:1 to take abemaciclib 200 mg monotherapy either; with a meal (Arm 1); in a modified fasting state defined as at least 1 h before or 2 h after a meal (Arm 2); or without regard to food (Arm 3). A meal was defined as whatever the patient would normally eat at that time. Patients were advised to avoid the consumption of grapefruit or grapefruit juice and other inducers and inhibitors of cytochrome P450 (CYP) 3A where possible, as these can affect the exposure of abemaciclib. Abemaciclib was administered on a continuous BID schedule with at least 6 h separating doses. Treatment cycles lasted 28 days. The study period consisted of the first 3 cycles of treatment for each individual patient and all results presented herein reflect the first 3 cycles only. Patients who continued to receive benefit following cycle 3 remained on treatment at investigator discretion and took abemaciclib without regard to food as per label. All study procedures were followed until study completion, which occurred when the last enrolled patient completed 3 cycles. Loperamide was the protocol specified anti-diarrheal medication. Patients received diarrhea management guidance per label and were provided with written support materials.

A training program using both face-to-face guidance and virtual media was developed to ensure standardized implementation of the e-diary globally. All patients completed a daily e-diary detailing the timing of each abemaciclib dose in relation to a meal, number of bowel movements (BM), and number of loperamide tablets. Supplemental Fig. 1 visualizes the patient’s e-diary experience and how they were prompted to respond within the e-diary. To determine an accurate baseline assessment, patients recorded daily number of BM for a week prior to study commencement. Adherence to study medication and e-diary was made available for investigator and site staff review.

PK samples were collected prior to first dose on cycle 1 day 1 and then in conjunction with other laboratory samples in cycle 1: day 15, cycle 2: days 1 and 15, and cycle 3: day 1.

### Objectives

The primary objective was to evaluate and summarize investigator-assessed incidence of Grade ≥ 3 and prolonged Grade 2 (> 7 days continuous duration) diarrhea; dose reductions, interruptions, and discontinuations due to diarrhea; and patient-reported utilization of anti-diarrheal medications during the first 3 cycles of treatment. Secondary objectives included overall safety, incidence and severity of TEAEs, serious adverse events, deaths and clinical laboratory abnormalities. PK analysis included steady-state concentrations of abemaciclib, its active metabolites LSN2839567 (M2) and LSN3106726 (M20), and total active analytes (sum of abemaciclib + M2 + M20). An exploratory objective was to evaluate and summarize incidence and duration of diarrhea reported daily by the patient using an e-diary.

### Statistical analysis

The study planned to enroll approximately 60 patients and was descriptive in nature. It was not powered for formal statistical comparison between groups, and sample size was based on regulatory guidance. Assignment to treatment arms were determined by a computer-generated random sequence and randomization was stratified by region.

Baseline analyses and patient dispositions were based on the intent-to-treat (ITT) population which included all patients enrolled and randomized in the trial. Safety analyses included all patients who received at least 1 dose of abemaciclib. Analyses of all primary investigator-assessed endpoints and adverse events were based on the safety population and summarized by study arms. Investigator assessments of diarrhea and dose modifications were required on day 1 of each cycle and were conducted as per their usual practice. While investigators and site staff had real time access to e-diary data to facilitate monitoring, ensure timely patient education, and for the grading of diarrhea, investigators were not required to review the e-diary data in their assessment of diarrhea. Adherence with study medication and e-diary was monitored and recorded by investigators. Patient data collected via the e-diary were summarized descriptively to provide daily estimates of loperamide use, compliance with assignment to food administration cohort, as well as incidence and duration of diarrhea, complementary to investigator assessment. PK analysis included all patients who had received at least 1 dose of abemaciclib and who had at least 1 evaluable PK sample.

## Results

### Patients

**Fig. 2:**
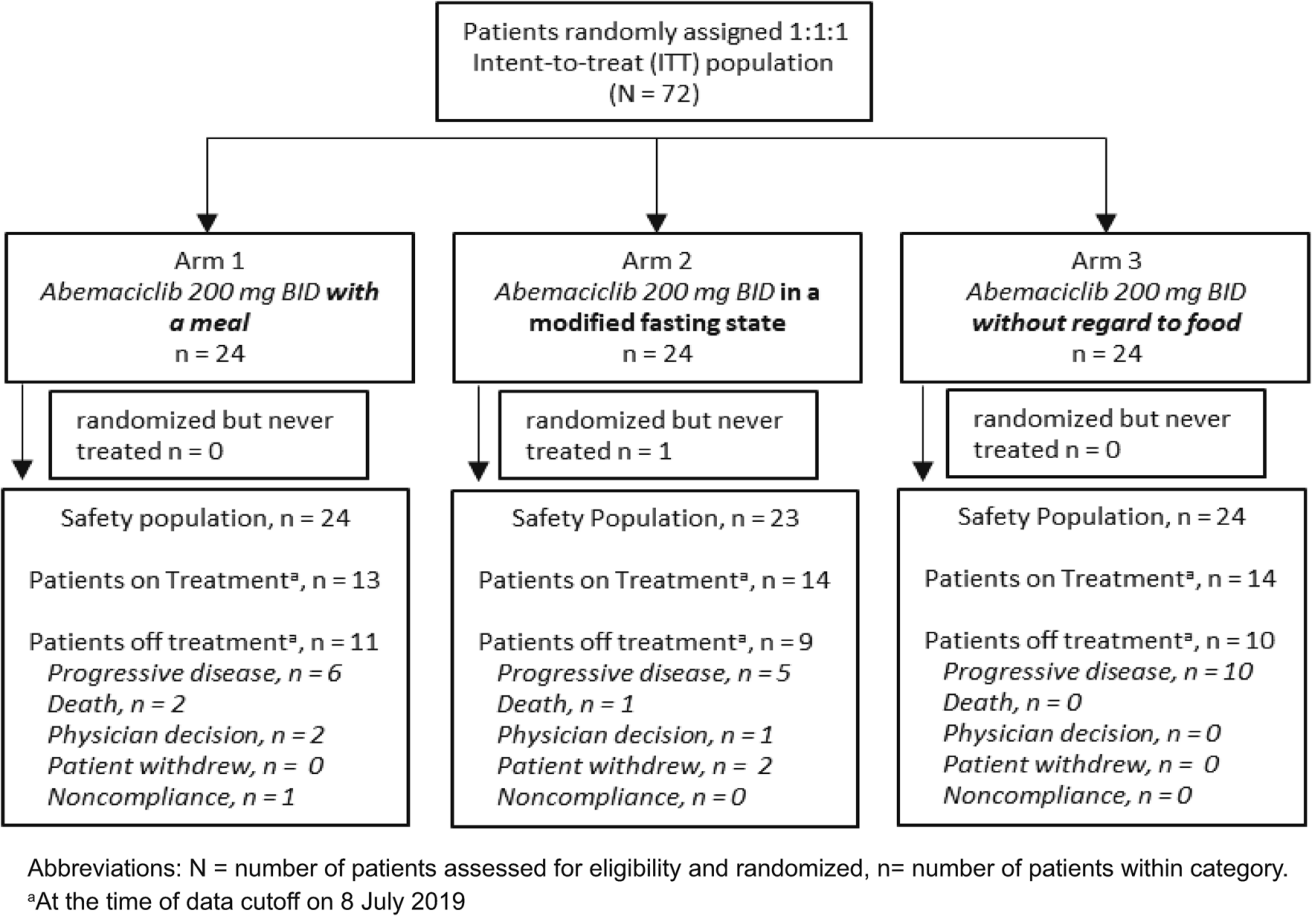
Consort diagram

Seventy-two patients were enrolled from December 4th, 2018 to April 10th, 2019 with 24 patients randomized to each of the 3 arms (Fig. 2). One patient in Arm 2 withdrew from the study and did not receive treatment, resulting in a safety population of 71 patients. Only 1 patient was male; this patient was randomized to Arm 1 (Table 1). The majority of patients (71%) were recruited from Australia, Spain, and Belgium. The mean age (years) was similar in Arm 1 (58), Arm 2 (57), and Arm 3 (58). Approximately 22% of patients had received ≥ 5 prior chemotherapies for ABC. Mean treatment compliance calculated by pill return across the 3 groups was 95.1%. Mean compliance with the assigned arm’s timing of abemaciclib dosing in relation to a meal was 97.4%. Mean e-diary completion compliance across all study arms was 95.7%.

**Table 1:** Demographics and baseline characteristics

	Overall *N* = 72	Arm 1 N = 24	Arm 2 N = 24	Arm 3 N = 24
Sex, *n* (%)				
Female	71 (98.6)	23 (95.8)	24 (100.0)	24 (100.0)
Male	1 (1.4)	1 (4.2)	0	0
Age categories, *n* (%)				
< 65 years	53 (73.6)	17 (70.8)	15 (62.5)	21 (87.5)
≥ 65 years	19 (26.4)	7 (29.2)	9 (37.5)	3 (12.5)
Age (years)				
Mean	57.6	58.2	56.9	57.6
Race, *n* (%)				
Asian	2 (2.8)	2 (8.3)	0	0
White	70 (97.2)	22 (91.7)	24 (100.0)	24 (100.0)
BMI, kg/m^2^ (SD)				
Mean	26.9 (4.5)	26.6 (4.3)	28.3 (5.4)	26.0 (3.6)
Region, *n* (%)				
Turkey/Russia	21 (29.2)	7 (29.2)	7 (29.2)	7 (29.2)
Australia/Spain/Belgium	51 (70.8)	17 (70.8)	17 (70.8)	17 (70.8)
ECOG Performance Status, *n* (%)				
0	37 (51.4)	13 (54.2)	10 (41.7)	14 (58.3)
1	34 (47.2)	11 (45.8)	13 (54.2)	10 (41.7)
Nature of disease, n (%)				
Visceral	59 (81.9)	20 (83.3)	17 (70.8)	22 (91.7)
Bone only	8 (11.1)	3 (12.5)	5 (20.8)	0
Others	4 (5.6)	1 (4.2)	1 (4.2)	2 (8.3)
Missing	1 (1.4)	0	1 (4.2)	0
Number of organ sites (%)				
1	14 (19.4)	3 (12.5)	8 (33.3)	3 (12.5)
2	24 (33.3)	9 (37.5)	5 (20.8)	10 (41.7)
≥ 3	33 (45.8)	12 (50.0)	10 (41.7)	11 (45.8)
Number of prior chemotherapy regimens for ABC, n (%)				
< 5	54 (75.0)	20 (83.3)	14 (60.9)	20 (83.3)
≥ 5	16 (22.2)	4 (16.7)	9 (39.1)	3 (12.5)

Intention-to-treat (ITT) population. All arms received 200 mg abemaciclib monotherapy twice per day: Arm 1 = taken with a meal; Arm 2 = taken without a meal (modified fasting condition); Arm 3 = taken without regard to food. *ECOG *Eastern Cooperative Oncology Group, *N *number of patients in the intent-to-treat population, *n *number of patients within category. ECOG status missing for 1 patient in Arm 2

Although only 2 of 18 investigators assessed the e-diary portal 3 or more times during the study period, the portal was accessed more frequently by study coordinators and nurses at each site. At the July 8th, 2019 data cutoff, 30 patients (41.7%) had discontinued treatment, with 21 patients (29.2%) discontinuing due to progressive disease.

### Diarrhea incidence and dose modifications

Food did not appear to impact incidence of investigator assessed diarrhea or dose modifications. While the numbers were low, the incidence of Grade ≥ 3 and prolonged Grade 2 diarrhea during the first 3 cycles of treatment were comparable across the 3 arms (Table 2). Notably, investigator assessed Grade 3 diarrhea occurred in only 1 patient overall (1.4%) during cycle 1 in Arm 1 and lasted 1 day. Prolonged Grade 2 diarrhea (> 7 days) occurred in 11 patients overall (15.5%).

**Table 2:** Primary endpoint results, investigator assessed

	Overall *N* = 71	Arm 1 *N* = 24	Arm 2 *N* = 23*	Arm 3 *N* = 24
Endpoint				
Diarrhea (any grade), n (%)	65 (91.5)	21 (87.5)	22 (95.7)	22 (91.7)
Grade 1 diarrhea, n (%)	35 (49.3)	10 (41.7)	14 (60.9)	11 (45.8)
Duration of Grade 1 diarrhea, median days	8	9	6	10
Grade 2 diarrhea, n (%)	29 (40.8)	10 (41.7)	8 (34.8)	11 (45.8)
Duration of Grade 2 diarrhea, median days	2	2	7	3
Grade 2 diarrhea lasting > 7 days, n (%)	11 (15.5)	2 (8.3)	4 (17.4)	5 (20.8)
Grade 3 diarrhea, n (%)	1 (1.4)	1 (4.2)	0	0
Duration of Grade 3 diarrhea, median days	1	1	0	0
Grade 4 diarrhea, n (%)	0	0	0	0
≥ 1 Dose reduction due to diarrhea, n (%)	9 (12.7)	4 (16.7)	2 (8.7)	3 (12.5)
≥ 1 Dose omission due to diarrhea, n (%)	7 (9.9)	4 (16.7)	1 (4.3)	2 (8.3)
Treatment discontinued due to diarrhea, n (%)	0	0	0	0
Loperamide use, n (%)	67 (94.3)	23 (95.8)	21 (91.3)	23 (95.8)

All arms received 200 mg abemaciclib monotherapy twice per day: Arm 1 = taken with a meal; Arm 2 = taken without a meal (modified fasting condition); Arm 3 = taken without regard to food. First three treatment cycles only. *N* = number of patients receiving at least 1 abemaciclib dose (safety population); *n* = number of patients within category

^*^one patient in Arm 2 discontinued from the study prior to receiving treatment

Dose reductions and omissions (dose interruptions) were balanced across Arms 1, 2, and 3 with 9 (12.7%) of all 71 patients having at least 1 dose reduction due to diarrhea and 7 (9.9%) of all patients requiring a dose omission for diarrhea (Table 2). 94% of patients used loperamide at least once in the first 3 cycles. Dose reduction due to fatigue and neutropenia were also balanced across all 3 arms, occurring in 4 (5.6%) and 8 (11.3%) of all 71 patients, respectively.

### Pharmacokinetics

**Fig. 3:**
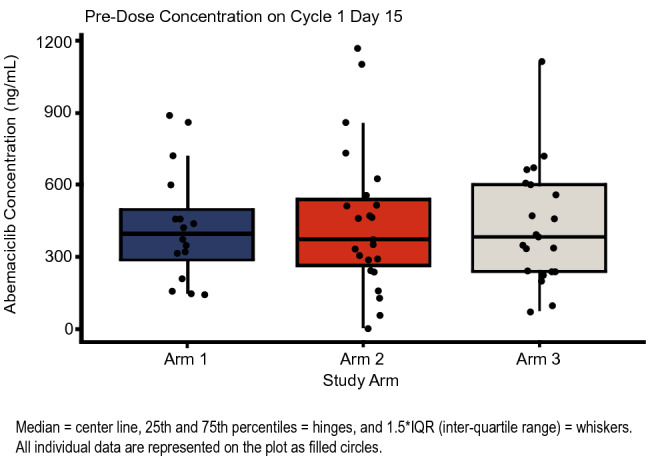
Abemaciclib concentrations on Cycle 1 Day 15 across the three treatment arms

A total of 69 patients had evaluable PK plasma samples: 24, 21, and 24 in Arms 1, 2, and 3, respectively. A total of 260, 256, and 259 samples were available for abemaciclib, and its active metabolites M2 and M20, respectively. Abemaciclib concentrations were comparable across the 3 treatment arms (Fig. 3), with average values ranging from 305 to 369 ng/ml on cycle 1 day 15. The mean range for total active analytes for the same time point was similarly comparable (1.38–1.60 uM).

### Treatment-Emergent adverse events (TEAEs)

Investigator-reported TEAEs of diarrhea were experienced by 21 patients (87.5%) in Arm 1, 22 patients (95.7%) in Arm 2, and 22 patients (91.7%) in Arm 3. No Grade 4 or 5 diarrhea events were reported (Table 2). TEAEs were otherwise consistent with prior abemaciclib trials and are listed in Table 3. No patients experienced a serious AE of diarrhea, or discontinued abemaciclib due to diarrhea, during the first 3 cycles. Importantly, despite the frequent use of loperamide, no patient experienced Grade 3 or greater constipation. Overall, 2 patients experienced Grade 1 constipation and 1 patient had a Grade 2 event. Two deaths occurred while on therapy or within 30 days of treatment discontinuation, both of which were associated with the study disease and assessed by the investigators to be unrelated to the study treatment. One death (4.2%) in Arm 1 was due to respiratory failure related to pulmonary progressive disease and pulmonary infection, and 1 death (4.3%) in Arm 2 was due to abnormal hepatic function where the patient had been diagnosed with liver metastasis prior to starting treatment. The most common treatment-emergent laboratory toxicities experienced by all patients included increased creatinine and decreased neutrophil count. Increases in serum creatinine levels are a known pharmacodynamic effect of abemaciclib caused by inhibition of renal tubular secretion without affecting glomerular function [10]. The TEAE of neutropenia has been observed across MONARCH studies. Neutropenia was not associated with severe infection [1, 3, 5].

**Table 3:** Treatment-emergent adverse events (TEAEs) Grade ≥ 3; investigator-assessed

	Overall *N* = 71	Arm 1 *N* = 24	Arm 2 *N* = 23	Arm 3 *N* = 24
TEAE	*n* (%)	*n* (%)	*n* (%)	*n* (%)
Neutropenia	20 (28.2)	7 (29.2)	3 (13.0)	10 (41.7)
Leukopenia	9 (12.7)	5 (20.8)	0	4 (16.7)
Thrombocytopenia	6 (8.5)	4 (16.7)	1 (4.3)	1 (4.2)
Nausea	4 (5.6)	1 (4.2)	3 (13.0)	0
Anemia	6 (8.5)	3 (12.5)	1 (4.3)	2 (8.3)
Lymphopenia	4 (5.6)	3 (12.5)	1 (4.3)	0
AST increased	4 (5.6)	3 (12.5)	1 (4.3)	0
ALT increased	2 (2.8)	2 (8.3)	0	0
Vomiting	3 (4.2)	1 (4.2)	2 (8.7)	0
Fatigue	4 (5.6)	1 (4.2)	2 (8.7)	1 (4.2)

All arms received 200 mg abemaciclib monotherapy twice per day: Arm 1 = taken with a meal; Arm 2 = taken without a meal (modified fasting condition); Arm 3 = taken without regard to food. First three treatment cycles only. Abbreviations: *ALT* alanine aminotransferase, *AST* aspartate aminotransferase, *N *number of patients receiving at least 1 abemaciclib dose (safety population), *n *number of patients within category. All events occurring in ≥ 2 patients in any one arm are included

^*^One patient in treatment Arm 1 died due to respiratory failure and one patient in Arm 2 died due to abnormal hepatic function; neither death was considered related to the study treatment

**Fig. 4:**
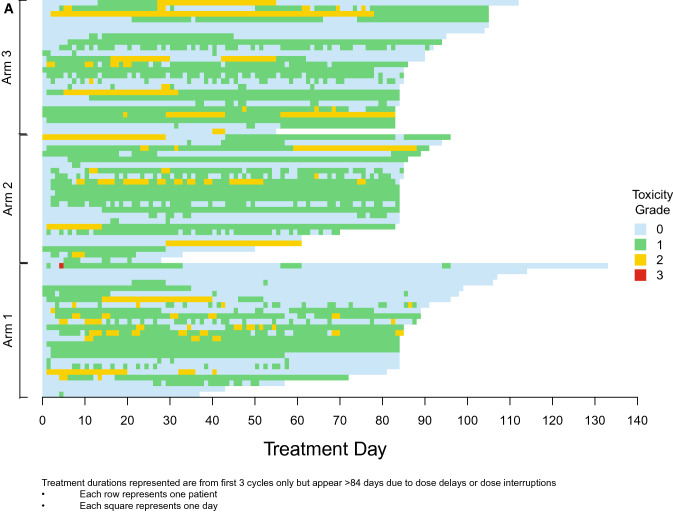

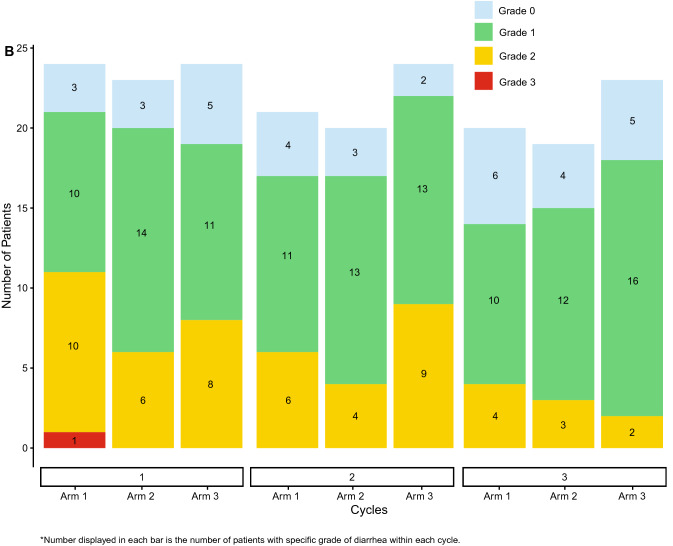

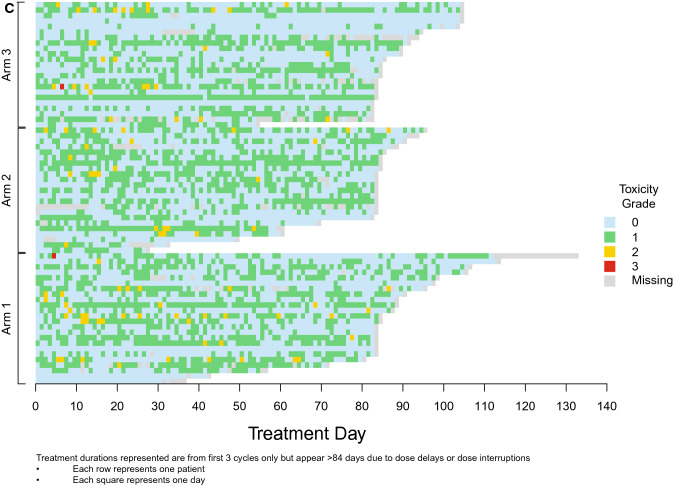

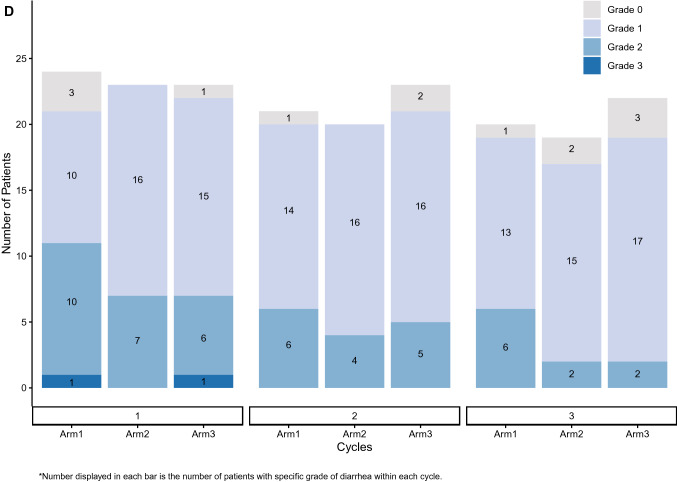

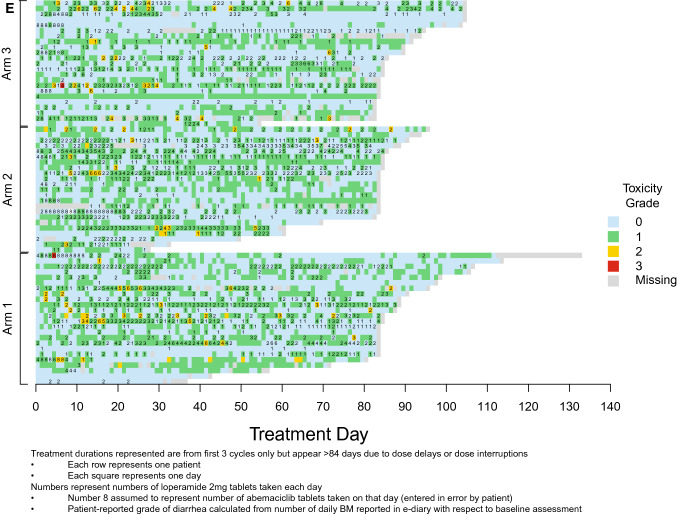
Duration and incidence of diarrhea in cycles 1–3. **A **Investigator assessed. **B** Investigator assessed. **C** Patient-reported e-diary. **D** Patient-reported e-diary. **E** Patient-reported e-diary account of loperamide doses in cycles 1–3

Diarrhea incidence and duration as assessed by the investigator (A and B) and the patient (C and D) during the first 3 cycles of therapy is illustrated in Fig. 4. This visually highlights the discrepancy between both assessments. One episode of Grade 3 diarrhea was reported by investigator assessment (for 1 patient in Arm 1) while 2 episodes were reported by patient assessment (1 patient each in Arm 1 and Arm 3). All reported Grade 3 episodes were of 1 day’s duration. Grade 2 diarrhea as assessed by the investigator was of longer duration than that assessed by the patient (median duration 2 days vs 1 day). Median duration of investigator-assessed Grade 2 diarrhea was 2 days overall: 2 days (Arm 1), 7 days (Arm 2), and 3 days (Arm 3). Finally, Fig. 4A demonstrates that Grade 1 diarrhea was assessed by the investigator as continuous while patient daily assessment via the e-diary (Fig. 4C) reported intermittent and short duration Grade 1 (See Fig. 4D).

Loperamide doses and corresponding patient-reported grade of diarrhea (calculated from daily numbers of BM relative to baseline recorded in the patient e-diaries) are displayed in Fig. 4E. While loperamide was used frequently, the number of tablets taken varied daily with the grade of diarrhea experienced.

## Discussion

Results from this trial demonstrate that food does not appear to impact the incidence or duration of prolonged Grade 2 and Grade ≥ 3 diarrhea, supporting the product label that abemaciclib may be taken with or without food. Abemaciclib PK was comparable across the 3 treatment arms, indicating no major differences in drug exposure related to meal timing. It has been reported that a high-fat, high-calorie meal increases abemaciclib plasma concentrations to a non-clinically meaningful extent [8]. However, the conditions of this trial simulate real-world drug administration rather than the high-fat, high-calorie meals in clinical studies specifically designed to assess the effect of food on PK parameters.

The findings reported here include several novel insights about abemaciclib-associated diarrhea beyond evaluation of the effect of food. Electronic capture of patient-reported outcomes (ePRO) can provide more granular reporting of adverse events than can be captured by traditional techniques [11]. Investigator assessments are complemented by daily patient reports of BM frequency and antidiarrheal use recorded with easy-to-use handheld e-diaries (Figs. 4A and B). Capturing daily patient-reported data via e-diary facilitated detailed, real-time characterization of the patient experience compared to retrospective physician assessment alone. Mean compliance with daily completion of the e-diary was 96% and is remarkable for a global trial in a patient population with heavily pre-treated ABC. This suggests that it is feasible, with appropriate training and oversight, to employ such tools in oncology trials. The overall incidence of diarrhea (any grade) in the current study was similar to that in MONARCH 1, where the dose used, and patient population were the same. However, the incidence of Grade 3 diarrhea was much lower here (1% vs 20%). This could suggest that as physicians have gained more experience with abemaciclib and incorporated standard diarrhea management guidelines into clinical care, abemaciclib-associated diarrhea is better understood and better managed. An assessment of the baseline number of BM as performed in this study would be important to allow an accurate grading of diarrhea as per CTCAE criteria. The median duration of Grade 2 and Grade 3 diarrhea, reported by both investigators and patients, was shorter than what was observed in the MONARCH 1, 2, and 3 trials [3–5]. Interestingly, patients in this study reported earlier resolution of Grade 2 diarrhea than investigators (Fig. 4). Furthermore, Fig. 4 illustrates that patients reported intermittent and shorter durations of Grade 1 diarrhea as opposed to the continuous Grade 1 diarrhea assessed by investigators. These data demonstrate the benefits of accurately recording grade and duration of diarrhea and reinforces the importance of recording the patient experience in real time. The additional granularity obtained through use of the daily e-diary permitted exploration of the relationship between antidiarrheal use and the incidence, grade, and duration of diarrhea. While loperamide was frequently used over the course of the study (Fig. 4C), the pattern of use suggests patients were appropriately educated regarding diarrhea management and varied their use of loperamide according to the severity experienced.

Consistent with management guidelines in the product label allowing for dose modifications for diarrhea, 12.7% of patients in this study had at least 1 dose reduction. A previous exploratory analysis demonstrated no difference in PFS outcomes for patients who dose reduced versus those who did not in the MONARCH 1, 2, and 3 trials, suggesting patients can receive a reduced dose and still derive meaningful benefit from abemaciclib treatment [12]. Those findings and the results of this study collectively suggest the standardized diarrhea management plan described in the product label appropriately manages abemaciclib-associated diarrhea without risking a decrease in efficacy.

This study had several limitations. First, it was of modest size limiting the ability to make comparisons between arms. Additionally, while data collected via e-diary was made available to site staff in real-time, e-diary user metrics reports showed it was predominantly accessed by study coordinators and nurses with only 2 of 18 investigators accessing the data ≥ 3 times in the 3-month study period (data on file). Ten of 18 investigators never accessed the e-diary data. This potentially explains the discrepancies seen between investigator and patient assessments (Fig. 4). Although high compliance rates with the daily patient e-diaries provided valuable insights into diarrhea incidence and management, it could be argued that patients may have been biased towards better management because of increased awareness and a daily requirement to enter data. Prior studies have indicated that active assessment and monitoring of symptomatic adverse events could yield positive outcomes for patients, including health related quality of life [13, 14]. Further studies are needed to understand the impact of patient self-monitoring on outcomes. Loperamide use was higher than what was previously seen in the MONARCH studies and it is possible that the high use of anti-diarrheal medication masked the impact of food on the incidence and severity of diarrhea. Food composition intake was also not standardized across groups. Finally, while PK analysis detected no discernable differences between the 3 arms, the study was not designed to assess the impact of food on abemaciclib PK, and it is likely that only large effects would be detected with a non-crossover study design and sparse PK sampling.

In conclusion, the results suggest that abemaciclib can be taken with or without food. Management guidance for associated diarrhea provided in the product label is appropriate. The current study results suggest that diarrhea is manageable with loperamide and dose modifications as needed. This is demonstrated by a progressive decrease in the frequency of Grade 3 diarrhea (in the first 3 cycles) for MONARCH 1, 2, and 3, and in the present study. Diarrhea onset is usually in the first cycle, and patient-reported e-diary data in the present study shows shorter duration of Grade 1 and 2 diarrhea than reported by investigator assessment. It is critical that patients receiving abemaciclib are educated on how to manage diarrhea and commence loperamide at the first sign of loose stools for optimal treatment benefit.

## Supplementary Information

Below is the link to the electronic supplementary material.

Supplementary file1 (DOCX 8190 KB)

## Data Availability

Eli Lilly and Company makes patient-level data available from Lilly-sponsored studies on marketed drugs for approved uses following acceptance for publication. Lilly is one of several companies that provide this access through the website clinicalstudydatarequest.com. Qualified researchers can submit research proposals and request anonymized data to test new hypotheses. Lilly’s data sharing policies are provided on the clinicalstudydatarequest.com site under the Study Sponsors page.
