# Supplementary material for: An open label, randomized phase 2 trial assessing the impact of food on the tolerability of abemaciclib in patients with advanced breast cancer
Source: Breast Cancer Res Treat. 2022 Aug 1;195(3):275–87. doi: 10.1007/s10549-022-06690-5 (PMC9464758; doi:10.1007/s10549-022-06690-5)

**An open label, randomized phase 2 trial assessing the impact of food on the tolerability of abemaciclib in patients with Advanced Breast Cancer**

Breast Cancer Research and Treatment

**Authors**: Elgene Lim^1*#^, Frances Boyle^2#^, Meena Okera^3^, Sherene Loi^4^, Sema Sezgin Goksu^5^, Gertjan van Hal^6^, Sonya C. Chapman^7^, Jonathon Colby Gable^8^, Yanyun Chen^8^, Gregory L. Price^8^, Anwar M. Hossain^8^, M. Corona Gainford^8^, Meritxell Bellet Ezquerra^9^

**Author Affiliations:** ^1^Garvan Institute of Medical Research, St. Vincent’s Clinical School, UNSW, NSW, Australia; ^2^Mater Hospital, North Sydney, NSW, Australia; ^3^Adelaide Cancer Centre, Kurralta Park, SA, Australia; ^4^Peter MacCallum Cancer Centre, Melbourne, VI, Australia; ^5^Akdeniz University Medical Faculty, Antalya, Turkey; ^6^Eli Lilly and Company, Utrecht, The Netherlands; ^7^Eli Lilly and Company, Windlesham, Surrey, UK; ^8^Eli Lilly and Company, Indianapolis, IN, USA; ^9^Hospital Universitario Vall d’Hebron and Vall d’Hebron Institute of Oncology (VHIO), Barcelona, Spain.

^#^ Contributed equally to the study

*** Corresponding Author:**

Elgene Lim

St. Vincent’s Hospital Sydney,

Kinghorn Cancer Centre, 370 Victoria Street,

Darlinghurst, NSW, Australia 2010

e.lim@garvan.org.au

**Supplemental Fig. 1 Patient facing e-diary**


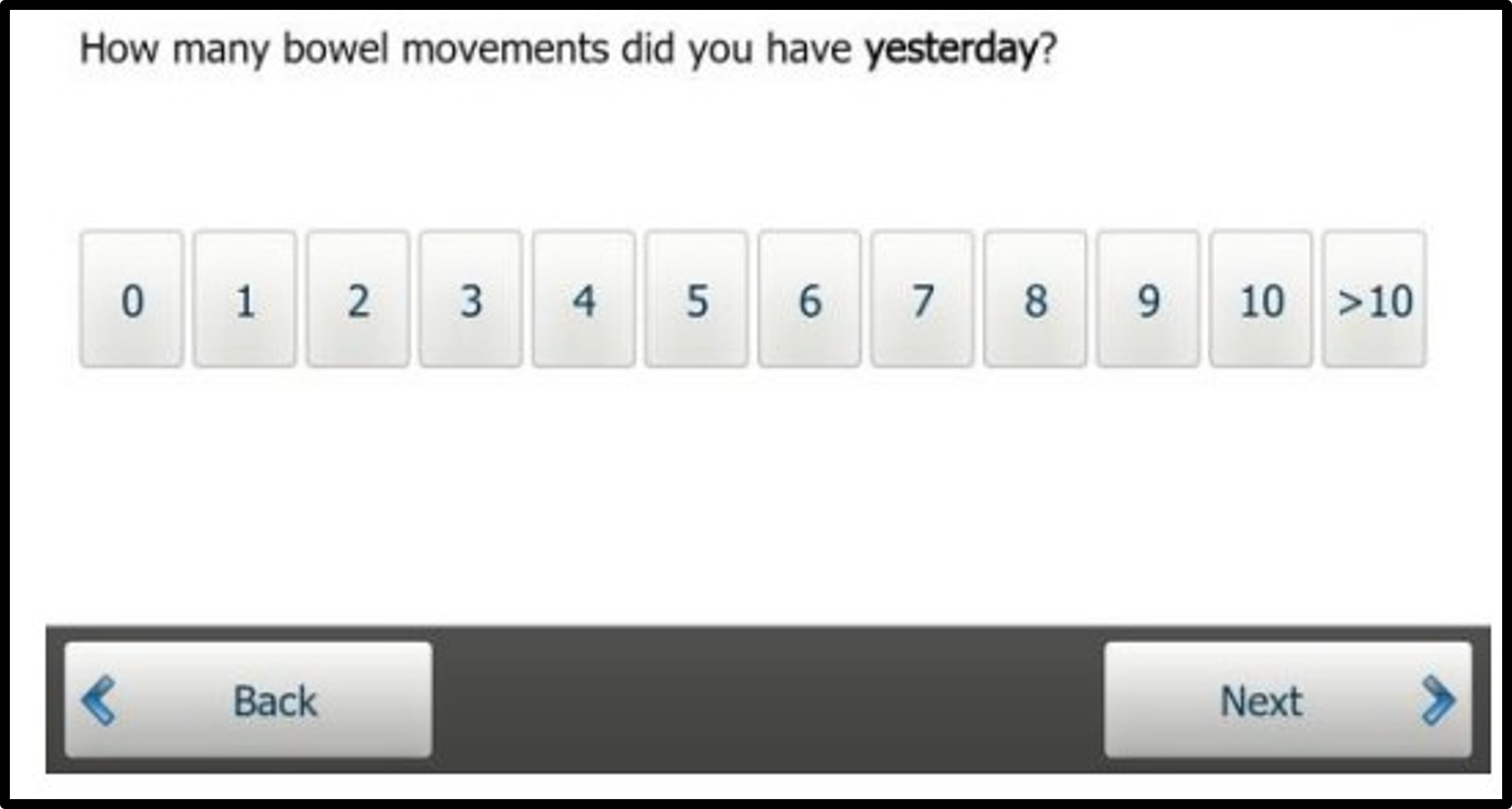

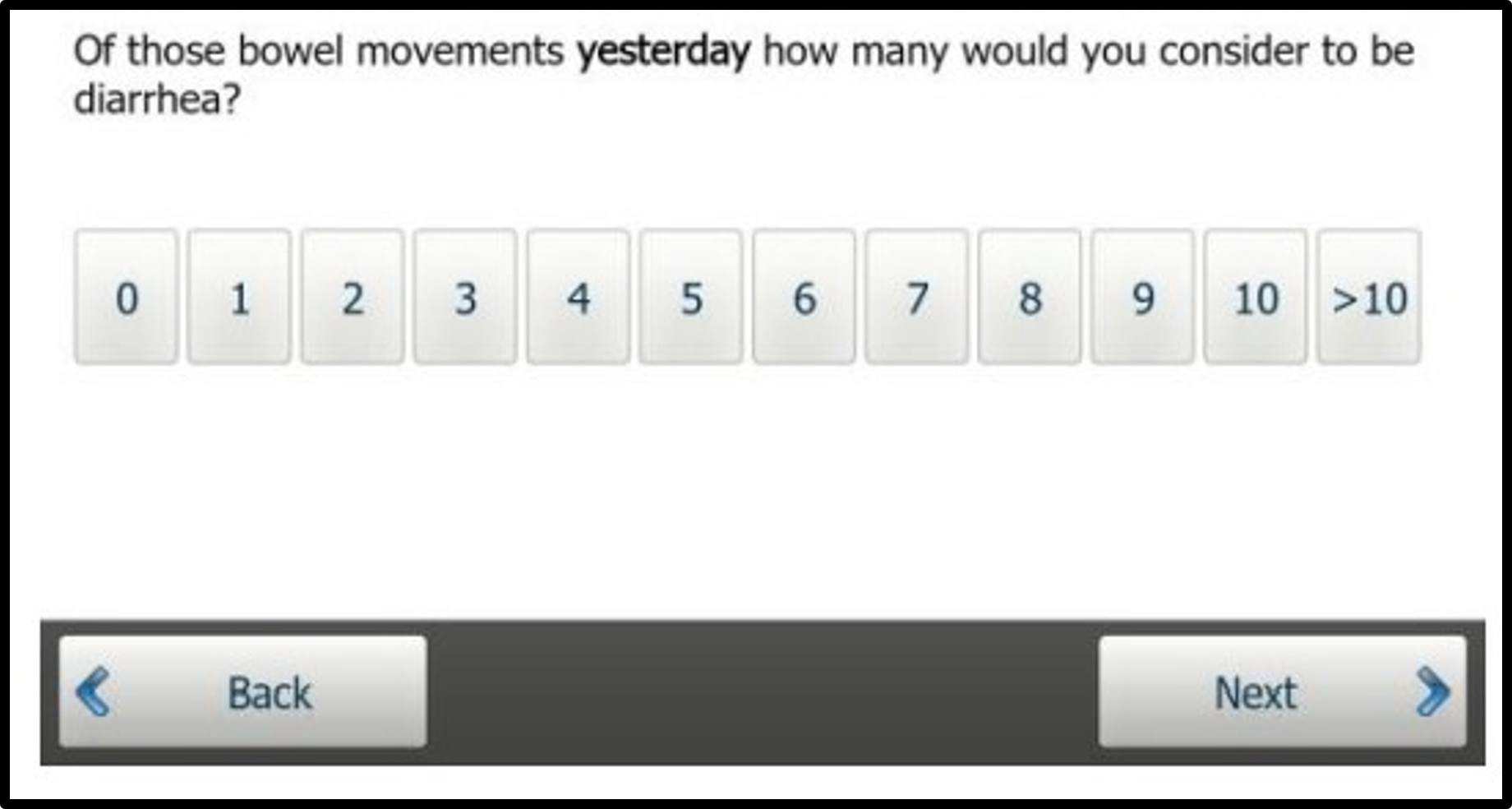

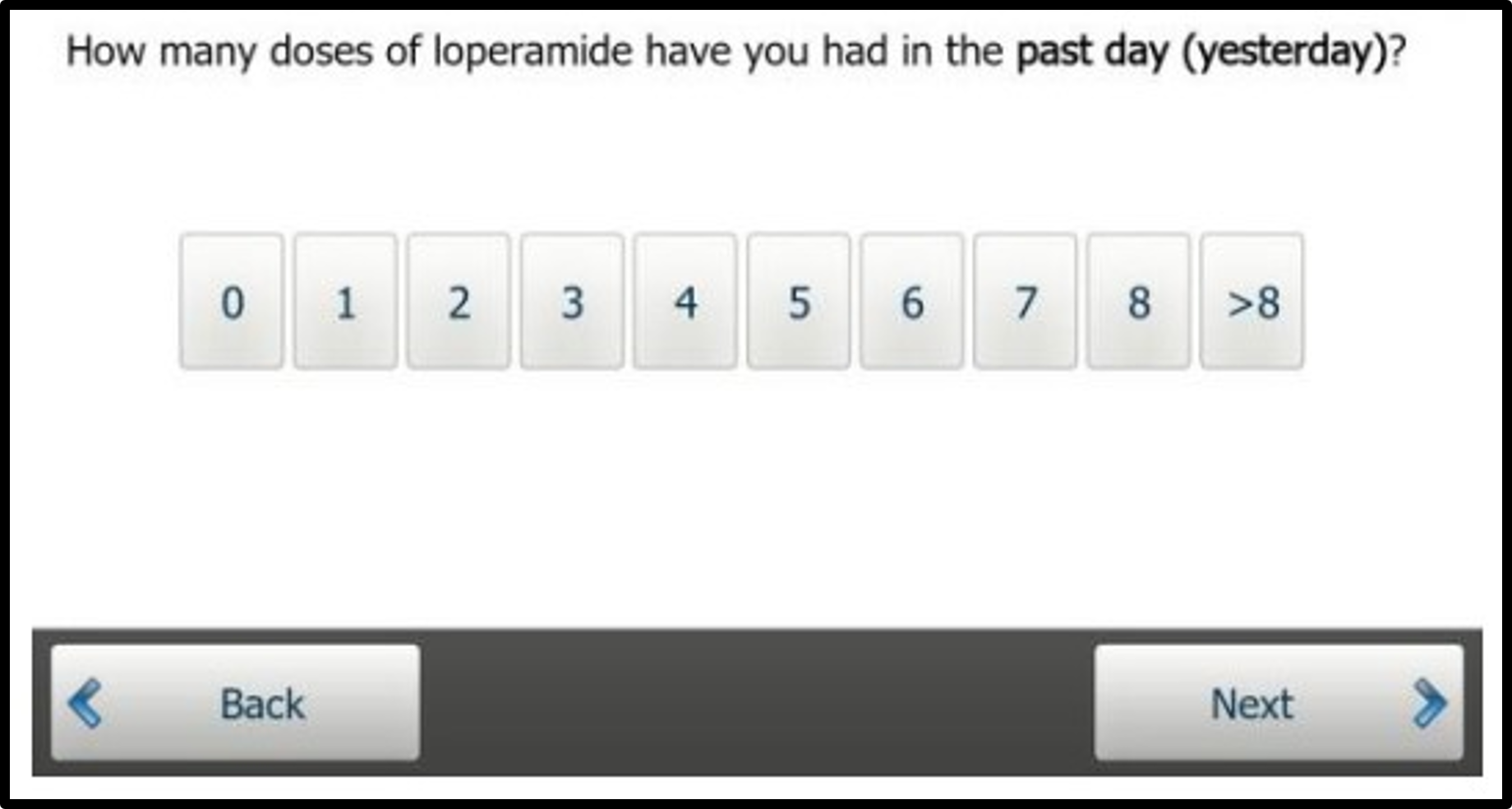


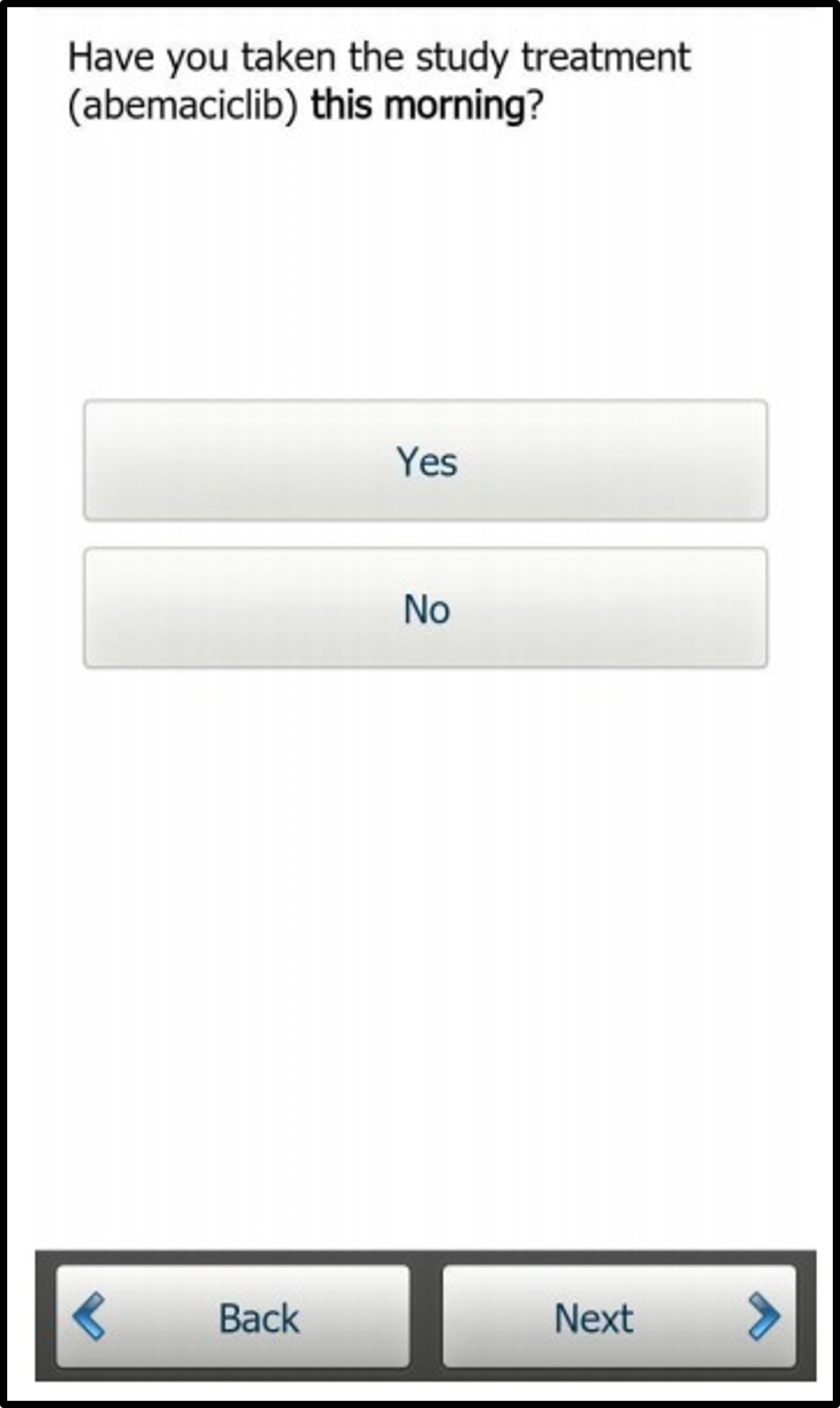

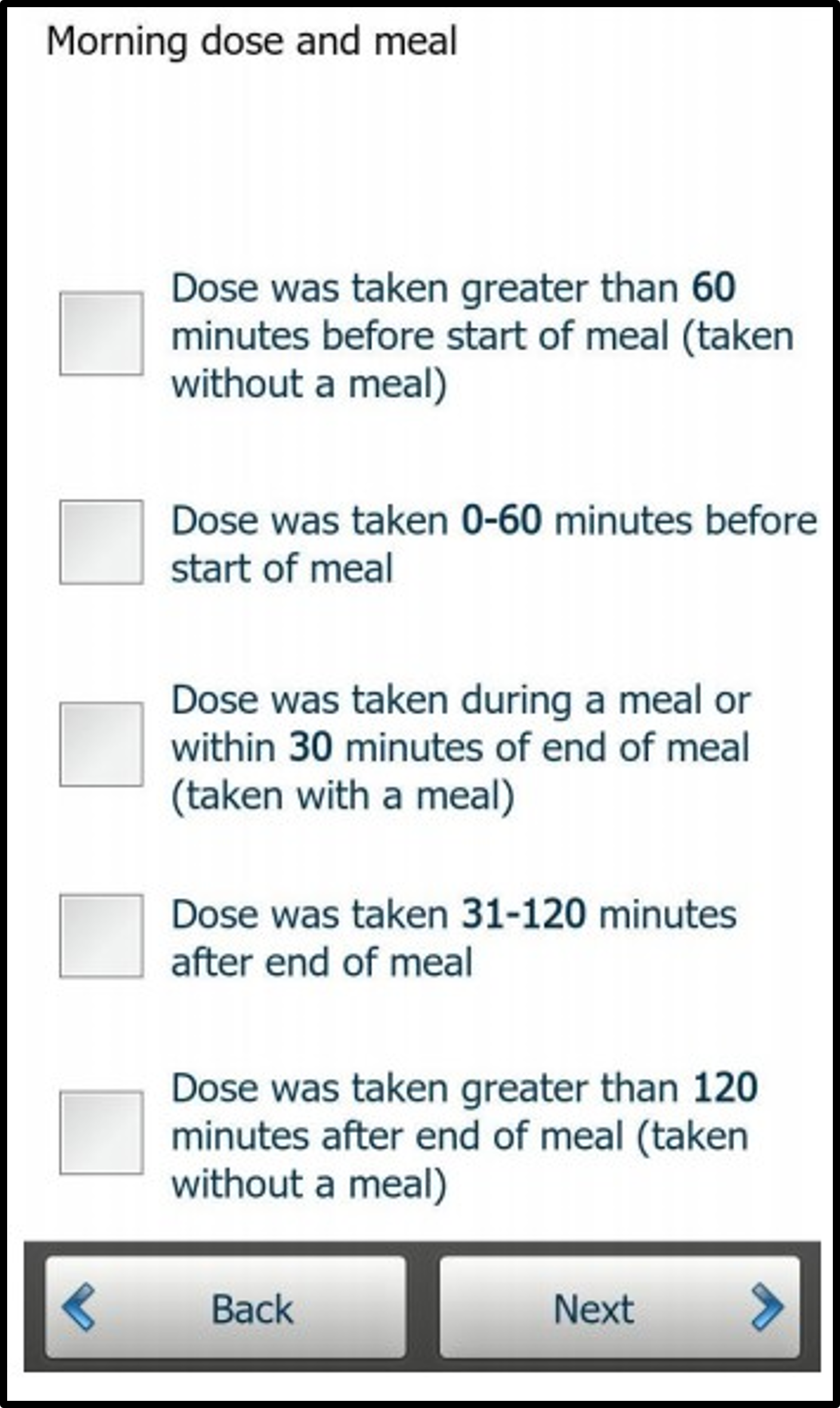

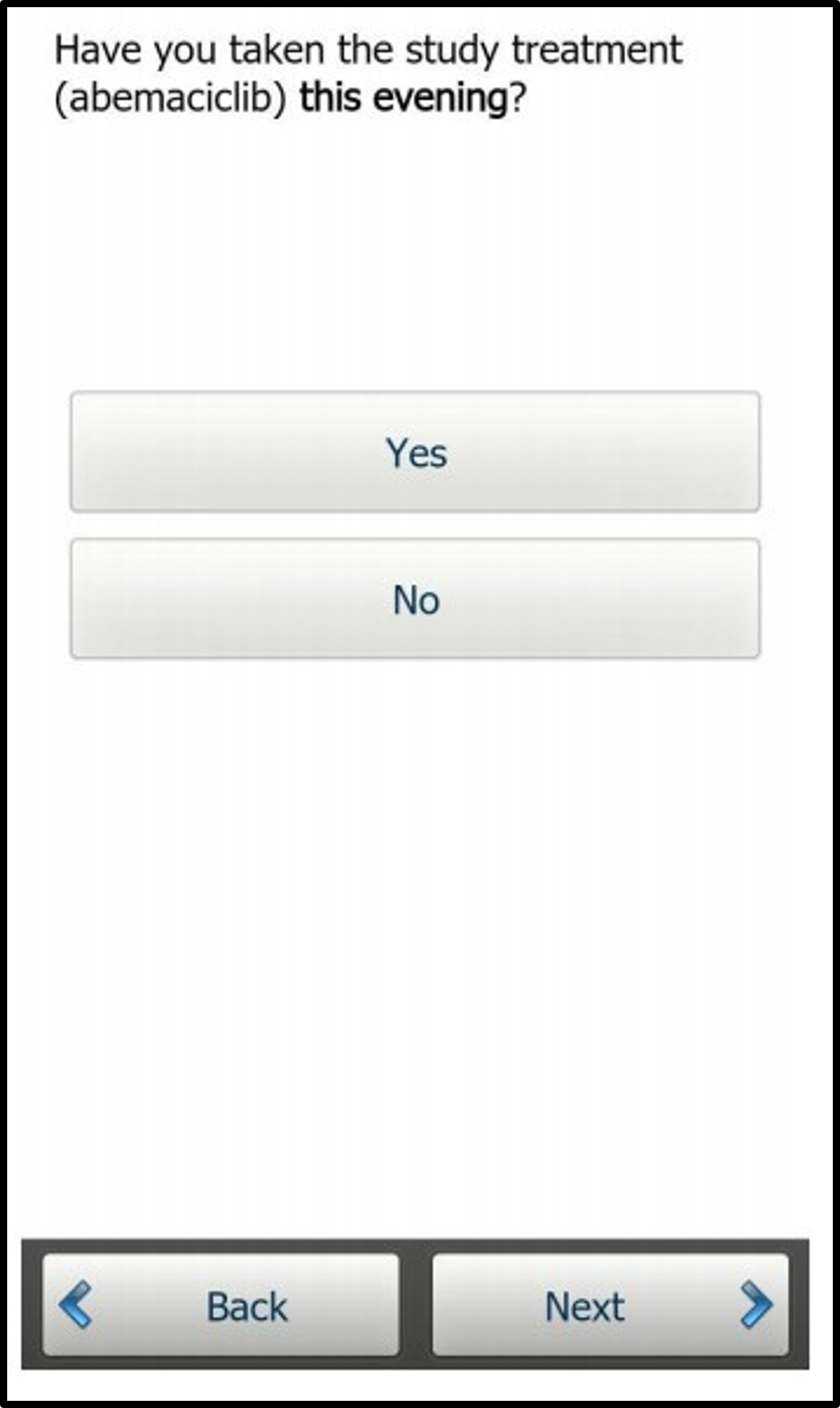

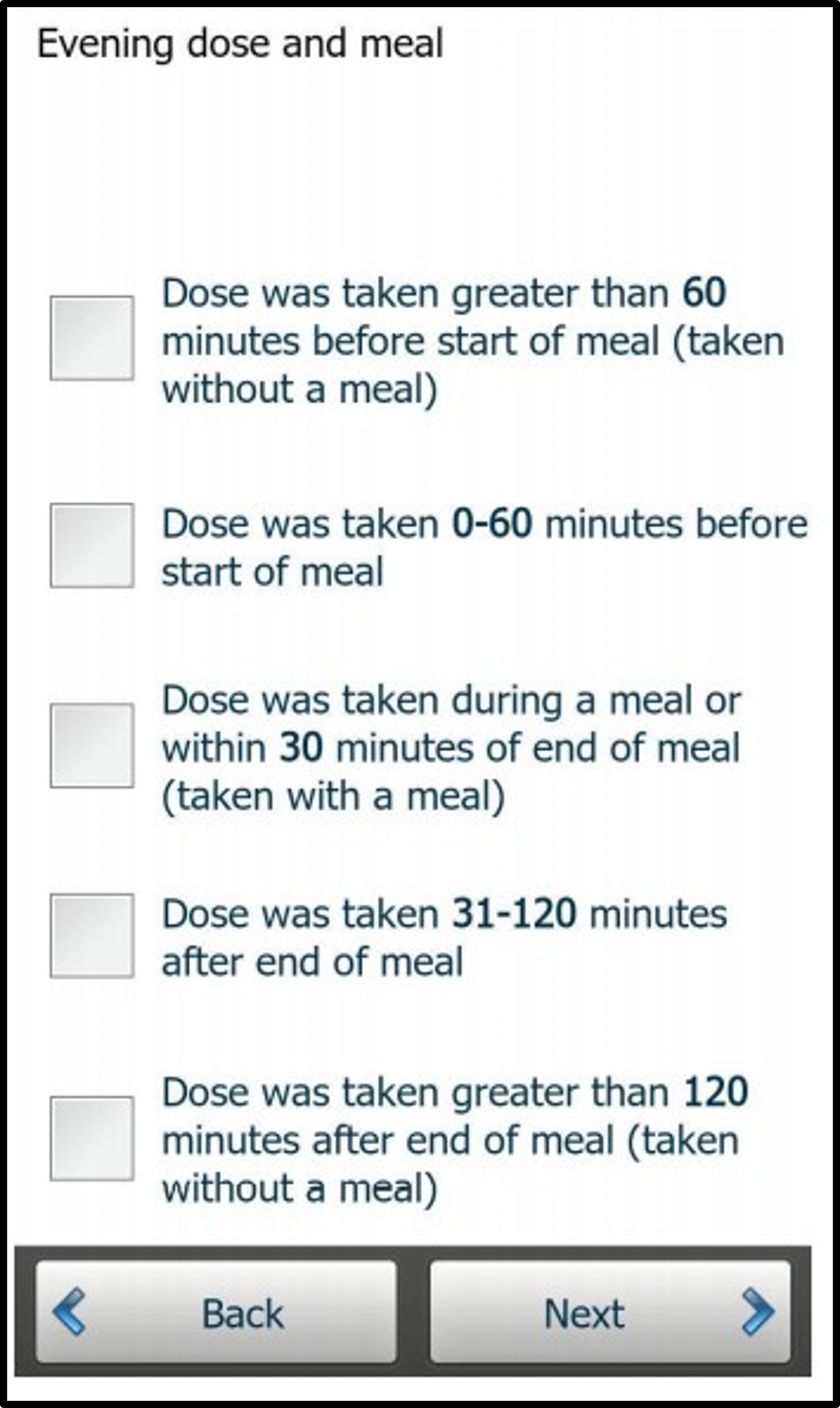

Supplement: Supplementary file 1 — Supplementary file1 (DOCX 8190 KB) [file 10549_2022_6690_MOESM1_ESM.docx]
